# Immune microenvironment evolution across the serrated neoplasia pathway and its relevance to immunotherapy

**DOI:** 10.3389/fonc.2026.1830436

**Published:** 2026-05-11

**Authors:** Chenfei Jin, Haoze Liu, Zejun Wu, Yuyuan Hu, Yipeng Cui, Renkai Guo, Huiyu Li

**Affiliations:** Third Hospital of Shanxi Medical University, Shanxi Bethune Hospital, Shanxi Academy of Medical Sciences, Tongji Shanxi Hospital, Taiyuan, China

**Keywords:** BRAF V600E, immune exclusion, immune microenvironment, immunotherapy, MLH1 methylation, serrated pathway, sessile serrated lesion

## Abstract

The serrated neoplasia pathway is a major molecular route to colorectal carcinogenesis, accounting for approximately 15%–30% of sporadic colorectal cancers and characterized by early BRAF V600E mutation, CpG island methylator phenotype, and distinct histopathologic features. Although immune checkpoint inhibitors have shown clear efficacy in MSI-H/dMMR colorectal cancer, serrated pathway–associated tumors remain highly heterogeneous in their molecular evolution and immune microenvironments. This heterogeneity may be associated with diverse immune phenotypes and potentially variable responsiveness to immunotherapy. Emerging evidence suggests that changes in the immune microenvironment may begin before invasive transformation. In sessile serrated lesions, early immune surveillance features, including intraepithelial CD8^+^ tissue-resident memory T cells, may be detectable before the development of a high tumor mutational burden state, whereas immune checkpoint upregulation and enrichment of regulatory immune populations indicate that adaptive immunosuppressive programs may arise in parallel. In this review, we synthesize evidence that immune alterations in serrated lesions are not merely accompanying features, but part of the biologic context in which lesions progress and later diverge. We therefore propose a stage-linked, hypothesis-generating framework in which early serrated lesions enter an immune-engaged yet counter-regulated state before later tending toward predominant inflamed MSI-H-like or immune-excluded MSS-like immune niches. Linking serrated lesions to changes in the immune microenvironment matters because it provides a biologic explanation for why morphologically similar precursor lesions may not be progression-equivalent and why advanced serrated-pathway cancers may diverge toward inflamed MSI-H-like or immune-excluded MSS-like states. Although not yet sufficient to alter classification or treatment selection, this perspective defines specific priorities for biomarker development, risk stratification, and mechanism-based therapeutic testing.

## 1 Introduction

Colorectal cancer (CRC) remains a major global malignancy (1). Traditionally, CRC has been viewed as arising predominantly through the classic adenoma–carcinoma sequence (2, 3). In recent years, however, accumulating evidence suggests that approximately 15%–30% of CRCs may develop via an alternative route, the serrated neoplasia pathway (4–6). This pathway originates from serrated premalignant lesions, including hyperplastic polyps (HPs), sessile serrated lesions (SSLs; previously referred to as sessile serrated adenoma/polyp, SSA/P) and SSLs with dysplasia (SSL-D), as well as traditional serrated adenomas (TSAs) (6–8). Among these entities, SSL is widely regarded as the most common and clinically important premalignant precursor in the serrated pathway; HPs are the most frequent benign serrated lesions, whereas TSAs are less common but still carry malignant potential (4, 6, 9). Histologically, serrated lesions are characterized by a “saw-tooth” architecture of the epithelium and glands (4, 6). Endoscopically, they are often flat and subtle with indistinct borders and a mucus cap; SSLs in the right colon, in particular, are prone to being missed, posing challenges for CRC screening and prevention (4, 10).

The serrated pathway is commonly initiated by an activating BRAF V600E mutation (with KRAS mutations observed in a minority of cases) and is frequently accompanied by a CpG island methylator phenotype (CIMP) (11–13). In SSLs, BRAF V600E is detected at a high frequency, and sustained MAPK signaling can disrupt crypt proliferation–differentiation homeostasis, giving rise to the characteristic serrated architecture (5, 12, 14). Meanwhile, BRAF-driven aberrant proliferation may be coupled with oncogene-induced senescence (OIS), a putative barrier to malignant progression in serrated lesions. By analogy with other BRAF-driven premalignant settings, this state may be accompanied by senescence-associated secretory programs capable of modulating the local microenvironment, although direct evidence in human serrated lesions remains limited (15–19).

These observations raise a central question: Do changes in the immune microenvironment merely accompany serrated progression, or do they help explain why serrated lesions are biologically heterogeneous and why later cancers diverge into distinct immune endpoints? We argue that this linkage matters because it reframes serrated lesions as progression-informative states, helps interpret later MSI-H-like versus MSS-like divergence, and identifies immune features that may become useful for biomarker development and therapeutic hypothesis generation.

## 2 Molecular and pathologic basis of the serrated pathway

The serrated pathway is best understood as a stepwise process in which distinctive clinicopathologic features reflect an underlying sequence of molecular events. Compared with the conventional adenoma–carcinoma pathway, serrated tumorigenesis is typically initiated by BRAF-driven epithelial reprogramming, followed by a period of constrained growth associated with oncogene-induced senescence, and then by progressive epigenetic and cooperative alterations that weaken this barrier and permit further progression. This sequence provides the molecular context for the later divergence of advanced serrated lesions toward either MLH1-methylated MSI-H/dMMR disease or MLH1-unmethylated MSS/pMMR disease, which is particularly relevant for understanding subsequent immune heterogeneity. In this way, the molecular progression outlined in this section provides the biologic premise for asking whether immune heterogeneity is also staged, rather than arising only after fully developed invasive disease.

### 2.1 Pathologic spectrum and endoscopic features: clinical relevance and missed-detection risk

In earlier clinical practice, serrated lesions were often considered benign or low-risk. Current consensus, however, recognizes serrated polyps as important precursors of sporadic CRC, with estimates suggesting that serrated pathway–related tumors account for approximately 15%–30% of sporadic cases (4, 20). Typical sessile serrated lesions (SSLs) are more frequently located in the right colon (10). On white-light endoscopy, they often appear flat or slightly elevated, similar in color to the surrounding mucosa, with an overlying mucus cap and indistinct margins (10). Their crypt openings may show a “cloud-like” or hazy pattern, making SSLs particularly prone to being missed during colonoscopic screening (10, 21–23). By contrast, conventional adenomas are usually more protuberant and easier to recognize, and therefore tend to have higher detection rates. Given the relatively high miss rate of serrated lesions, they are considered an important contributor to interval colorectal cancer—CRC diagnosed after a negative colonoscopy and before the next recommended examination—and warrant heightened clinical attention (24).

### 2.2 Differences in initiating events: APC-driven versus BRAF-driven tumorigenesis

Mechanistically, most sporadic CRCs arise through the conventional adenoma–carcinoma sequence, which is typically initiated by loss of adenomatous polyposis coli (APC) function and followed by acquisition of additional driver alterations, including KRAS, TP53, and SMAD4 (2), that promote progression from adenoma to invasive carcinoma (25, 26). In contrast, the serrated pathway is less commonly initiated by APC alterations and is more often driven by early BRAF V600E mutation (11, 27). In a prospective study, BRAF mutation was detected in 78% of sessile serrated adenomas, but was rare in conventional adenomas, supporting BRAF mutation as a key early event in serrated tumorigenesis (12). This distinction in initiating events is important because it sets the stage for a different sequence of epithelial, epigenetic, and microenvironmental changes from those seen in the conventional adenoma–carcinoma pathway.

### 2.3 The proliferation–senescence sequence after BRAF activation: MAPK signaling, metabolic reprogramming, and the OIS barrier

In *in vivo* models, the BRAF V600E mutation leads to sustained activation of the MAPK pathway, which induces marked proliferation of the intestinal crypt epithelium and can generate disorganized serrated architectures in mice (19, 28). Notably, BRAF-driven early proliferation does not necessarily progress directly to malignant transformation. This phase is often followed by oncogene-induced senescence (OIS), characterized by upregulation of p16^INK4a, which may place lesions into a relatively quiescent premalignant state (18, 19). Senescence is often accompanied by a senescence-associated secretory phenotype (SASP), in which senescent cells release inflammatory mediators and chemokines that can reshape the local microenvironment and potentially facilitate later progression (29, 30). More recent functional studies using BRAF-driven serrated neoplasia models and transcriptomic analyses suggest that cholesterol biosynthesis and broader metabolic reprogramming are highly active in these lesions (14). Rather than being purely epiphenomenal, these metabolic programs may help sustain crypt hyperproliferation and lesion cell survival, raising the possibility that metabolic pathways, including cholesterol metabolism, could represent therapeutic entry points for intervention in serrated lesions (14). The key question, therefore, is not only how BRAF-driven lesions initiate, but how they escape this early constrained state and acquire the additional alterations required for progression.

### 2.4 Epigenetic drivers and escape from the OIS barrier: CIMP accumulation, CDKN2A silencing, and cooperative alterations

Against a BRAF-mutant background, SSLs tend to exhibit a CpG island methylator phenotype (CIMP), which is more pronounced in progressed SSLs with dysplasia (SSL-D), particularly in areas of high-grade dysplasia (31, 32). Progressive accumulation of CIMP is associated with advancement from SSL to dysplasia and carcinoma and may contribute to this process through epigenetic silencing of tumor suppressor genes and DNA repair genes (13, 31). Mechanistic studies indicate that, as lesions evolve, promoter methylation of CDKN2A can increase and lead to loss of p16^INK4a expression, suggesting that epigenetic inactivation of p16 is an important step in overcoming the OIS barrier described above (18). In addition, BRAF activation alone is often insufficient to drive efficient malignant progression and typically requires cooperative events—such as dysregulation of cell-cycle control (e.g., p16 silencing or TP53 inactivation), aberrant TGF-β signaling, reactivation of WNT signaling (e.g., alterations in RNF43 or truncating APC mutations), or emergence of MMR deficiency (28, 33–35). Collectively, these alterations may weaken early growth constraints and are associated with progression toward high-grade dysplasia and invasive carcinoma (35, 36). Together, these cooperative alterations provide the molecular basis for the later divergence described in the next section, particularly the transition toward MLH1-methylated MSI-H/dMMR states or MLH1-unmethylated MSS/pMMR states.

### 2.5 Predominant late progression trajectories: MLH1 hypermethylation-associated MSI-H versus MLH1-unmethylated MSS states

After serrated lesions develop dysplasia, MLH1 promoter hypermethylation often occurs, leading to loss of MLH1 protein expression and, consequently, mismatch repair deficiency with an MSI-H phenotype (35, 37). This event has been widely viewed as an important molecular transition during serrated pathway carcinogenesis (27, 35). Available data indicate that a substantial proportion of SSL-D cases show MLH1 loss within dysplastic areas, supporting a close link between MLH1 silencing and this stage of progression (38, 39). In BRAF-mutant SSL-D, MLH1 methylation–associated silencing is more commonly associated with progression toward MSI-H CRC, whereas lesions without MLH1 methylation are more likely to progress to MSS CRC (35, 40). Accordingly, two predominant late-stage trajectories are often described within the serrated pathway: BRAF-mutant/MLH1-methylated lesions tending toward MSI-H/dMMR states and BRAF-mutant/MLH1-unmethylated lesions tending toward MSS/pMMR states (27, 35).

**Figure 1:**
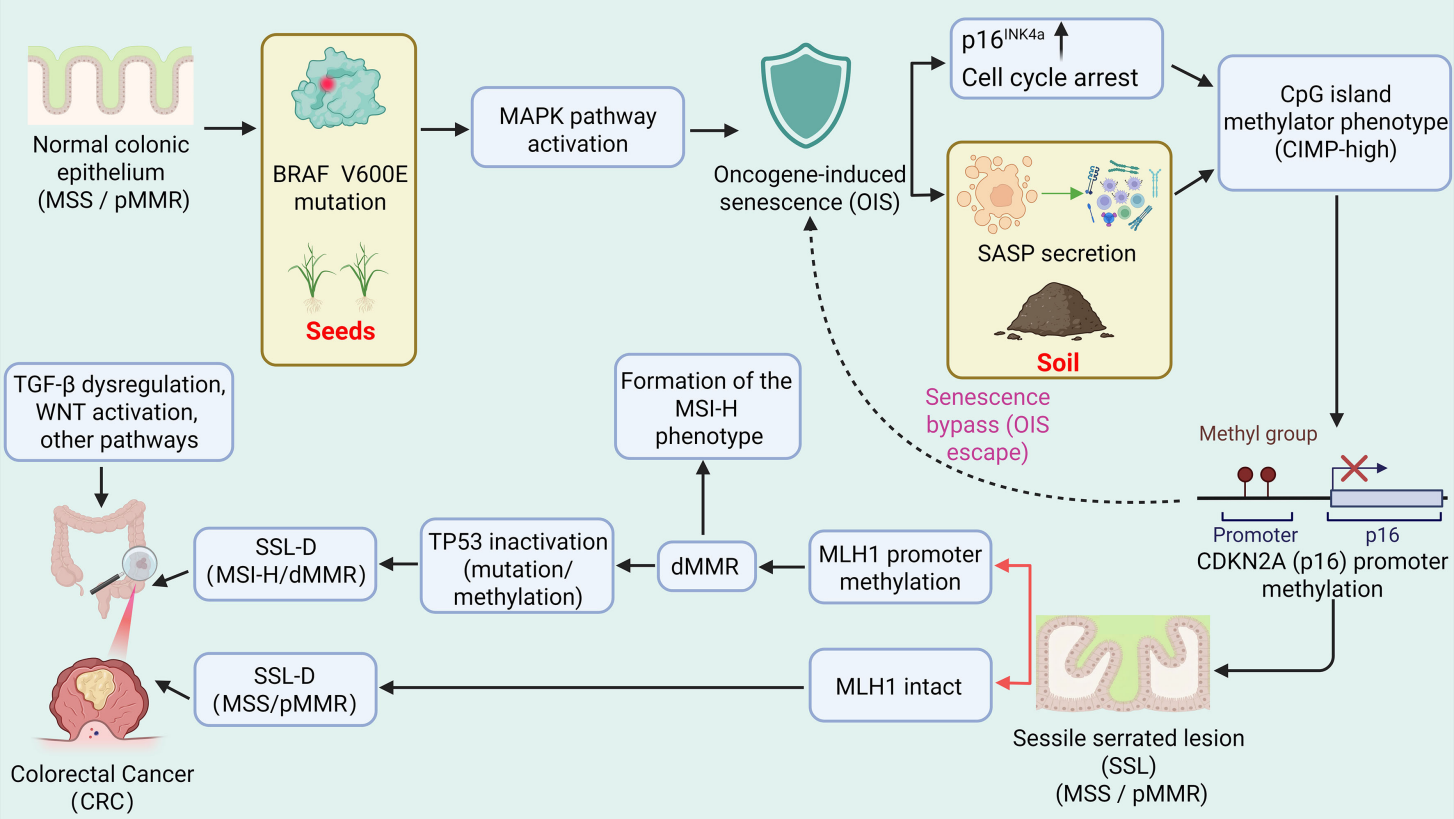
Molecular framework of serrated pathway progression and predominant late trajectories toward MSI-H/dMMR and MSS/pMMR states. This schematic summarizes the proposed molecular evolution of the serrated neoplasia pathway. Early BRAF V600E mutation activates MAPK signaling and promotes formation of sessile serrated lesions (SSLs), while oncogene-induced senescence (OIS) may act as an early barrier to malignant progression. Progressive CpG island methylator phenotype (CIMP-high) and promoter methylation events may facilitate escape from this barrier. At the dysplastic stage, lesions may diverge into two major routes: one with MLH1 promoter methylation, leading to dMMR/MSI-H progression, and another with preserved MLH1, leading to MSS/pMMR progression through additional cooperative alterations. This figure summarizes a conceptual framework rather than a strictly linear sequence for all serrated lesions.

**Figure 1** illustrates the molecular evolution of the serrated neoplasia pathway and its predominant late divergence toward MSI-H/dMMR and MSS/pMMR states.

## 3 Immune microenvironment evolution along the serrated pathway

This section addresses a central question: how should immune changes be interpreted across the serrated neoplasia continuum? Current evidence suggests that early serrated lesions are not simply immune-cold, but often display both immune engagement and regulatory restraint, with later progression tending toward distinct endpoint immune niches (41–43). We therefore examine this pattern by integrating mechanistic links, empirical observations, and the limits of current inference, with the aim of clarifying the biologic plausibility of a stage-linked immune framework.

### 3.1 Mechanistic links between epithelial drivers and immune phenotypes along the serrated pathway

Although the available evidence remains incomplete, several molecular events in serrated tumorigenesis can be linked, at least conceptually, to distinct immune phenotypes. This mechanistic framework helps explain three broad observations: first, the early immune engagement and counter-regulatory features observed in BRAF-driven serrated lesions; second, the tendency of MLH1-methylated lesions to culminate in inflamed MSI-H/dMMR-like states; and third, the evolution of a subset of MLH1-unmethylated BRAF-mutant tumors toward stromal- and myeloid-dominant immune exclusion. Importantly, this framework should be interpreted as an integration of current evidence rather than as proof of a deterministic pathway (27, 35, 42–45).

#### 3.1.1 BRAF/MAPK–OIS/SASP–early immune microenvironment changes

Early BRAF V600E activation is a defining initiating event in the serrated pathway and is already linked to epithelial proliferation, oxidative-stress responses, and oncogene-induced senescence-associated states in premalignant lesions (18, 19, 43). A plausible consequence of this BRAF/MAPK-driven context is the emergence of a mixed immune landscape, in which increased immune-cell recruitment coexists with early immunosuppressive or counter-regulatory mechanisms (29, 43). Single-cell analyses of serrated lesions have shown that SSLs harbor enhanced cytotoxic CD8^+^ T-cell activity, largely attributable to an increased proportion of CD103^+^CD8^+^ tissue-resident memory T cells (TRM), while immunosuppressive populations, including regulatory T cells, anti-inflammatory macrophage-like cells, and PDGFRA^+^ fibroblasts, are already detectable and become more prominent in SSL-D (43). These findings argue against a purely immune-cold premalignant niche and instead suggest that changes in immune composition and function accompany early oncogenic progression in serrated lesions. However, because these data are largely derived from cross-sectional single-cell and spatial analyses, they should not be interpreted as direct evidence of coordinated parallel evolution between the immune landscape and epithelial progression (42, 43). Retinoic-acid-related programs may contribute to TRM differentiation and maintenance, based on experimental studies of intestinal CD103^+^CD8^+^ TRM biology; however, this link remains indirect in human serrated lesions (46). Taken together, currently available data indicate that BRAF-driven serrated lesions can already show immune-cell recruitment together with emerging regulatory and suppressive features early in their evolution (19, 43), even before overt hypermutation or invasive carcinoma develops.

#### 3.1.2 CIMP/MLH1 methylation–dMMR/MSI-H–inflamed but restrained phenotype

A second mechanistic axis links progressive epigenetic remodeling to the inflamed MSI-H/dMMR endpoint. In serrated lesions, CIMP accumulation and subsequent MLH1 promoter hypermethylation are well-established molecular events associated with progression to dMMR/MSI-H disease in a substantial subset of dysplastic lesions (27, 35, 37, 40). The best-supported immune consequence of this transition is not that methylation itself directly instructs the microenvironment, but rather that progression to dMMR/MSI-H increases mutational burden and neoantigen generation, thereby favoring stronger immune recognition, lymphocytic infiltration, and checkpoint induction (41, 47–49). This inference is supported primarily by observed associations in MSI-H/dMMR colorectal cancer (47–49). By contrast, data showing that MAPK suppression can augment intratumoral CD8^+^ T-cell infiltration in BRAFV600E colorectal cancer should be viewed as mechanistically relevant but not as direct proof of the same process in serrated precursor lesions (45). Taken together, these observations support the interpretation that MSI-H/dMMR serrated tumors are often inflamed yet functionally restrained, while still allowing for alternative explanations rooted in epithelial evolution (35, 47, 50–52).

#### 3.1.3 TGF-β, stromal/myeloid programs, and immune exclusion in the MSS branch

By contrast, the MLH1-unmethylated BRAF-mutant branch appears more compatible with an immune-excluded MSS/pMMR phenotype shaped by cooperative tumor-intrinsic, stromal, and myeloid programs. The current molecular framework already identifies WNT reactivation and aberrant TGF-β signaling as cooperating events in serrated progression, and available preclinical work provides a mechanistic basis for linking these pathways to immune exclusion (33–35, 44). In organoid and precursor-lesion models, TGF-β signaling is already active in sessile serrated adenoma-like lesions and can direct BRAFV600E serrated precursors toward a mesenchymal CMS4-like state, rather than the apoptotic response typically observed in conventional adenomas (44). This is relevant because mesenchymal/TGF-β-high programs are commonly associated with stromal expansion, reduced immune permissiveness, and treatment resistance (44, 53). In parallel, stromal remodeling in serrated lesions includes accumulation of PDGFRA^+^ fibroblasts, while broader CAF-rich tumor models show that fibroblast abundance can produce a CD8^+^ T-cell-excluded phenotype and reduce sensitivity to immune-checkpoint blockade (43, 53). When integrated with evidence for CXCR2-associated myeloid recruitment and reduced antigen-presentation/IFN-γ responsiveness in BRAF-mutant/MSS CRC, these features are best interpreted as co-occurring processes that together support immune exclusion, rather than as a fully resolved causal sequence (43, 44, 53–55). Because direct lineage-matched evidence in human serrated tumors remains limited, this MSS branch should still be presented as a predominant mechanistic trajectory rather than a fully proven sequence.

### 3.2 What is established versus what is inferred

Several elements of the proposed framework are supported by direct evidence, whereas others remain inferential. At present, relatively well-supported findings include: (i) early BRAF-driven serrated tumorigenesis with frequent CIMP accumulation (27, 32, 35); (ii) the association of MLH1 methylation with progression toward dMMR/MSI-H disease in a substantial subset of dysplastic serrated lesions (35, 37, 40); and (iii) evidence that premalignant serrated lesions can already exhibit immune-cell infiltration, checkpoint upregulation, and spatial changes in the immune microenvironment (41–43). Together, these observations indicate that immune alterations are already detectable before invasive carcinoma develops.

What remains unresolved is the causal interpretation of these findings. Current evidence does not establish that early immune features directly determine later immune trajectories, nor does it show that immune regulation itself drives the transition toward MSI-H-like and MSS-like immune contexts. Rather, the proposed framework should be regarded as an interpretive synthesis derived mainly from current cross-sectional evidence (35, 41, 44). Longitudinal sampling, lineage-resolved studies, and functional experiments will be required to determine which observed immune changes are causal, correlative, or epiphenomenal.

Supporting evidence from animal and preclinical models is currently stronger for epithelial and epigenetic progression than for immune causality. In murine serrated-neoplasia models, prolonged oncogenic Braf signaling is sufficient to induce progressive DNA methylation changes over time, supporting an epithelial-driven route to epigenetic evolution (19, 28). Additional models suggest that BRAF-mutant serrated progression, particularly toward MSS-like disease, may involve cooperation with pathways such as WNT and TGF-β signaling (28, 33, 34, 44). By contrast, direct experimental evidence that early immune signals induce MLH1 methylation, or that immune regulation itself determines later MSI-H versus MSS divergence, remains limited. For this reason, the value of the proposed framework is not that it resolves causality at present, but that it helps define which progression-related, biomarker-related, and translational questions can now be asked more explicitly and tested more directly.

### 3.3 A hypothesis-generating model of serrated immune pre-programming and divergent trajectories

We propose a hypothesis-generating model that summarizes the stage-associated immune patterns described above. It is intended to organize cross-sectional observations rather than to establish a causal or deterministic sequence (41–43). Its function is therefore interpretive: to provide a structured way of understanding how early immune changes in serrated lesions may relate to later divergence into distinct immune endpoint states.

#### 3.3.1 Stage I: premalignant immune priming/pre-programming (primarily corresponding to SSL)

In this model, SSLs occupy an early stage in which immune recognition is already evident but is accompanied by inhibitory feedback, including checkpoint expression and the emergence of suppressive immune populations (41–43). Hallmarks include TRM/IEL enrichment together with emerging checkpoint expression and suppressive immune populations (42, 43). Whether this state contributes to later divergence or mainly accompanies epithelial progression remains unresolved.

#### 3.3.2 Stage II: a proposed transition toward divergent immune trajectories (mainly corresponding to SSL-D)

At the dysplastic stage, MLH1 promoter methylation should first be regarded as a well-established molecular transition in serrated progression (35, 37, 40). Its association with immune divergence is supported mainly by cross-sectional differences between MLH1/MSI-defined groups rather than by direct evidence that methylation itself drives changes in immune composition and function (41–43).

An alternative explanation is that epithelial progression, including epigenetic evolution and MLH1 silencing, primarily determines later MSI status, with immune engagement arising largely as a downstream consequence of increased mutational and neoantigen burden (19, 28, 35). Within this framework, MLH1-methylated lesions more often align with inflamed MSI-H/dMMR-like states, whereas MLH1-unmethylated BRAF-mutant lesions more often align with immune-excluded MSS-like states, although this should be interpreted as an integrative model rather than a directly proven lineage-resolved sequence (35, 41, 44). The importance of this proposed transition is that it offers a framework for interpreting why serrated precursor lesions with superficially similar morphology may later diverge into biologically and immunologically distinct outcomes.

#### 3.3.3 Stage III: predominant endpoint immune niches (invasive carcinoma stage)

Within this framework, two broad endpoint immune contexts can be summarized as follows:

The MSI-H/dMMR-like endpoint is typically more infiltrated and checkpoint-high, consistent with an inflamed but regulated state, although immune-evasion mechanisms such as antigen-presentation loss or impaired IFN-γ signaling may still limit response (48–52). By contrast, the BRAF-mutant/MSS-like endpoint is more often characterized by immune exclusion, myeloid suppression, and limited sensitivity to ICI monotherapy, which has motivated investigation of rational combination strategies (44, 56–58).

Key differences between these two predominant endpoint immune contexts are summarized in **Table 1**.

**Table 1:** Conceptual comparison of two predominant endpoint immune trajectories in serrated pathway-associated colorectal carcinogenesis.

Dimension	MSI-H/dMMR (often associated with BRAF mutation and MLH1 methylation)	BRAF V600E/MSS (MLH1 unmethylated)
Key molecular transition	MLH1 promoter methylation → loss of MLH1 expression → dMMR/MSI-H	No MLH1 methylation-associated transition; pMMR/MSS is maintained
Basis of immunogenicity	Higher tumor mutation burden and neoantigen load, with stronger immune recognition	Relatively limited neoantigenicity; immune recognition is weak or constrained
Dominant immune niche	Inflamed but restrained	Immune-excluded and myeloid-dominant
Immune infiltrate profile	More abundant TILs/IELs and CD8^+^ T cells; more active checkpoint pathways	Prominent suppressive myeloid/neutrophil-like populations and immunosuppressive macrophages; restricted effector T-cell entry
Spatial immune organization	More often an infiltrated/inflamed microenvironment	More often an excluded/barrier microenvironment, with T cells retained in stroma or at the invasive margin
Checkpoint phenotype	PD-1/PD-L1 and related pathways are often upregulated, consistent with inflammation-associated adaptive immune regulation and inhibitory feedback	Checkpoint upregulation may occur, but does not necessarily indicate effective antitumor immunity; responses are often spatially constrained or functionally unproductive
Major immune-evasion mechanisms	Antigen-presentation defects (for example, MHC-I pathway loss) and IFN-γ pathway alterations contributing to primary or acquired resistance	Myeloid suppressive programs, impaired antigen presentation, and stromal immune exclusion
Clinical behavior	In biomarker-defined MSI-H/dMMR CRC, strong benefit from ICIs is established, although primary and acquired resistance still occur	Generally poorer prognosis; limited benefit from conventional therapies; low response rates to ICI monotherapy
Current therapeutic responsiveness	Robust clinical evidence supports benefit from PD-1 monotherapy and PD-1 + CTLA-4 combinations, although most studies are stratified by MSI-H/dMMR rather than serrated origin	Limited and heterogeneous evidence; treatment often relies on combination strategies involving targeted-induced immune remodeling plus ICI amplification
Representative therapeutic strategies	ICI-centered approaches, with additional strategies to overcome resistance, including dual checkpoint blockade or immunomodulatory combinations	Targeted therapy (BRAF/EGFR ± MEK) and/or microenvironment remodeling as a backbone, combined with ICI and/or anti-myeloid strategies
Translational biomarker directions	MSI/dMMR status, TIL density, and markers of antigen presentation or IFN-γ signaling	Myeloid chemotactic axes (for example, CXCR2-related networks), spatial immune-exclusion metrics, and stromal/immune-barrier features

This table summarizes a conceptual framework derived from current pathological, molecular, and immune profiling studies. Clinical implications summarized here should be interpreted primarily in relation to validated biomarkers such as MSI/dMMR rather than as direct proof of serrated-origin-specific therapeutic effects.

MSI-H, microsatellite instability-high; dMMR, deficient mismatch repair; MSS, microsatellite stable; pMMR, proficient mismatch repair; TILs, tumor-infiltrating lymphocytes; IELs, intraepithelial lymphocytes; ICI, immune checkpoint inhibitor.

#### 3.3.4 Exceptions, mixed phenotypes, and transitional immune states

Although the proposed framework highlights two predominant immune trajectories, real serrated lesions and serrated pathway–associated cancers are unlikely to follow a strictly binary or deterministic course (42, 43). Mixed phenotypes may occur across molecular and histologic stages, including lesions with overlapping immune engagement and suppression, focal dysplastic heterogeneity, collision-like features, or spatially compartmentalized immune programs (38, 39, 43). Likewise, transitional states may exist in which MSS lesions display partial inflammatory features or MSI-H lesions retain elements of immune exclusion (41, 44). These exceptions are important, because they caution against overinterpreting the model as a fixed branching hierarchy. Instead, the MSI-H-like and MSS-like states are better viewed as predominant endpoint tendencies emerging from a heterogeneous continuum (42, 43).

#### 3.3.5 Testable predictions

Rather than serving as a descriptive staging scheme alone, this model yields three testable implications concerning progression, immune divergence, and therapeutic vulnerability. First, TRM/IEL-rich SSLs are expected to display a reproducible immune-engaged but regulated state, in which evidence of immune engagement coexists with checkpoint upregulation and suppressive immune circuitry (41–43). Second, progression to SSL-D, particularly in lesions acquiring MLH1 methylation, is expected to be accompanied by changes in spatial immune organization and in programs related to antigen presentation and IFN-γ responsiveness (40, 42, 43, 59, 60). Third, BRAF-mutant/MSS tumors are expected to show relatively consistent myeloid- and stromal-associated exclusion signatures that may help define biomarker-informed combination strategies (44, 56, 58). These should be regarded as hypotheses for prospective testing rather than as established temporal or clinical relationships.

Rather than merely organizing existing observations, the model therefore identifies measurable features that can be tested in future studies to determine whether early immune states help distinguish lesions with different progression routes and different translational relevance.

**Figure 2:**
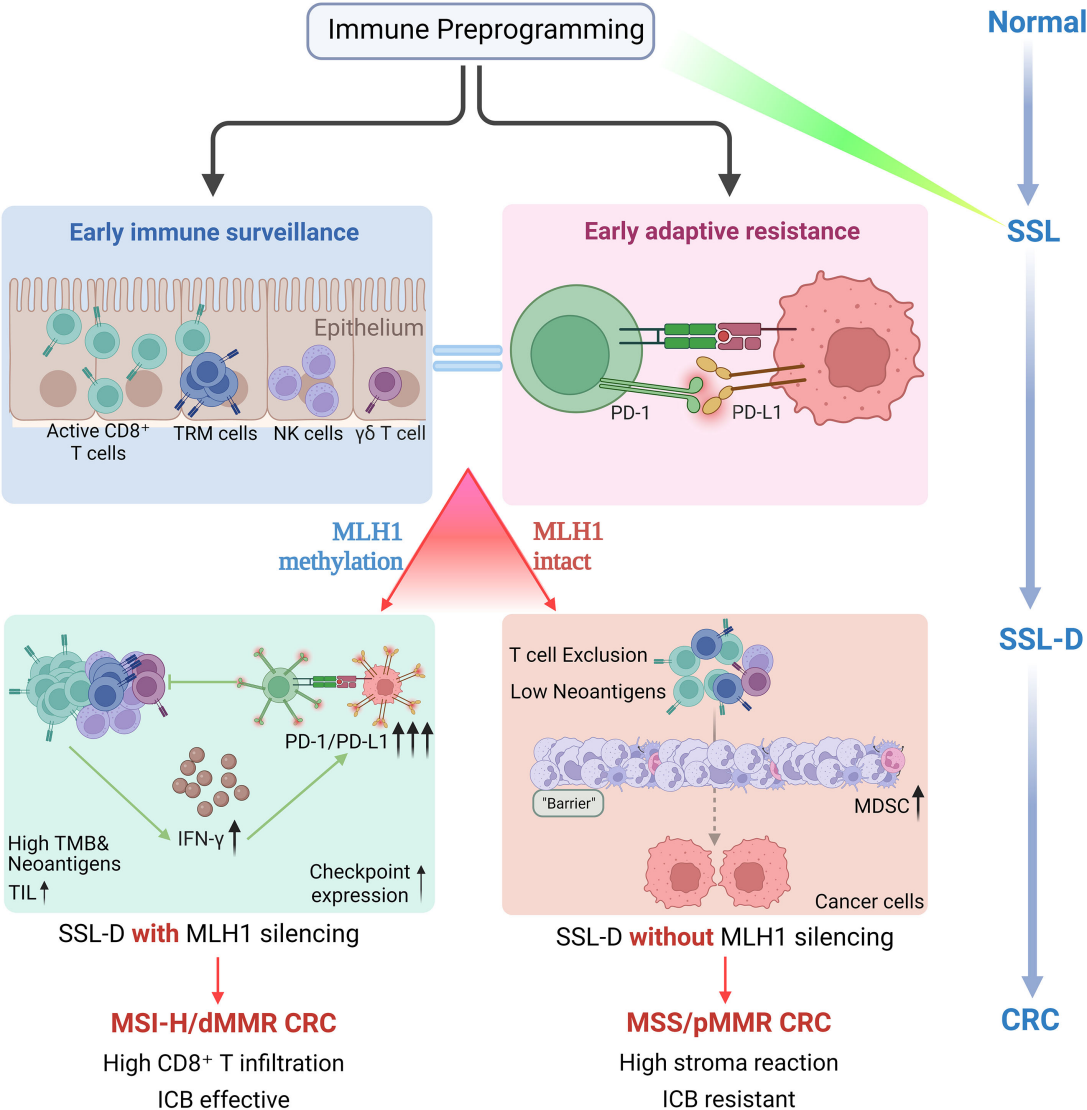
Proposed immune pre-programming and divergent trajectory model along the serrated neoplasia pathway. This schematic illustrates a hypothesis-generating model of changes in the immune microenvironment across the serrated pathway. At the SSL stage, early immune surveillance may coexist with adaptive immune restraint, including infiltration of CD8^+^ T cells, TRM cells, NK cells, and γδ T cells, together with early PD-1/PD-L1 upregulation. At the SSL-D stage, lesions may diverge toward predominant trajectories according to MLH1 status. MLH1 silencing is proposed to favor a predominant MSI-H/dMMR-like immune trajectory, whereas lesions without MLH1 silencing may remain MSS/pMMR and develop a more immune-excluded, myeloid-dominant context.

A schematic overview of this hypothesis-generating immune pre-programming and divergent trajectory model is provided in **Figure 2**.

### 3.4 Methodological and biological sources of heterogeneity in immune readouts

Interpretation of immune microenvironment changes across serrated lesions requires caution because multiple biological and technical variables can materially influence reported immune phenotypes. These include anatomic location, lesion size, the proportion and spatial distribution of dysplastic epithelium, sampling depth, inflammatory background, histologic classification, and assay platform. For example, right-sided lesions may differ from distal lesions in baseline immune tone and molecular context (61, 62); small or superficially sampled lesions may underrepresent stromal or invasive-margin compartments; and different analytic platforms such as conventional immunohistochemistry, multiplex immunofluorescence, single-cell sequencing, and spatial transcriptomics capture distinct aspects of immune organization. In addition, MSI status itself may vary according to the method used for assessment, including immunohistochemistry, PCR-based testing, or sequencing-based approaches (63–65). These sources of heterogeneity likely contribute to variability across studies and should be considered when comparing immune-cell densities, checkpoint expression, and inferred immune states (41–43).

Importantly, many comparisons across lesion stages are derived from different patients rather than serial sampling of the same lesion lineage (42, 43). Inter-patient variability in genetics, epigenetic background, inflammatory context, microbiome composition, and host immunity therefore remains a major confounder, and apparent cross-stage immune differences should therefore not be read as direct evidence of temporal progression.

To facilitate interpretation of apparent discrepancies across studies, the major biological and methodological sources of heterogeneity that may influence immune readouts are summarized in **Table 2**.

**Table 2:** Key sources of heterogeneity affecting immune readouts across studies of serrated lesions and serrated pathway-associated colorectal cancer.

Source of heterogeneity	Representative example	Likely effect on immune readouts	Implication for cross-study comparability
Anatomic location	Proximal/right-sided versus distal/left-sided lesions	May shift baseline immune tone and molecular context; proximal serrated-enriched lesions may more often show MSI-related and checkpoint-associated features	Cohorts with different proportions of proximal versus distal lesions may report different IEL, CD8^+^, PD-L1, or MSI-associated immune features even within the same nominal lesion category
Lesion size and dysplastic fraction	Small nondysplastic SSL versus large SSL with focal or extensive dysplasia	Larger or more dysplastic lesions may appear more inflamed, more checkpoint-positive, or more spatially remodeled because they contain more advanced epithelial change	Comparisons across cohorts may be confounded if lesion size or the proportion of dysplasia is not matched or clearly reported
Sampling depth/analyzed compartment	Superficial biopsy, whole-polyp section, epithelium-focused analysis, stromal or invasive-margin assessment	Superficial or epithelium-biased sampling may overemphasize IELs while undercapturing stromal, myeloid, or exclusion-related architecture	Immune readouts are not directly comparable unless the analyzed compartment is specified, because epithelial, lamina propria, stromal, and invasive-margin signals may differ substantially
Histologic classification and diagnostic reproducibility	HP versus SSL; SSL versus SSL-D; need for serial sections; collision lesions	Misclassification can dilute or distort apparent immune differences between categories	Apparent biological inconsistency across studies may partly reflect pathology classification differences rather than true immune divergence
Inflammatory background of the mucosa	Background colitis, reactive mucosal inflammation, local injury	Can increase lymphocyte or myeloid cell density independent of serrated neoplastic biology and may overestimate lesion-specific immune engagement	Studies that do not control for inflammatory background may exaggerate immune microenvironmental changes attributed to serrated lesions
Assay platform	Conventional IHC, multiplex immunofluorescence, single-cell RNA-seq, spatial transcriptomics	Different platforms capture different layers of biology: cell density, co-expression, cell state, or spatial organization	Discordance between studies may reflect measurement modality rather than true biological contradiction
Marker selection and scoring method	CD3 versus CD8 versus PD-1/PD-L1; IEL count per 200 epithelial cells versus semiquantitative score	Different markers and scoring frameworks may yield different apparent trends in “immune engagement” or “checkpoint upregulation”	Cross-study synthesis is limited if marker panels, cutoffs, and scoring systems are not harmonized
MSI/MMR assessment method	MMR IHC, PCR-based MSI testing, NGS-based MSI calling	Method-dependent subgroup assignment may alter which lesions/tumors are labeled MSI-H/dMMR or MSS/pMMR	Studies stratified by different MSI/MMR methods are not fully interchangeable when comparing immune features across molecular subgroups

These factors may influence reported differences in IEL density, CD8^+^ infiltration, checkpoint expression, spatial immune organization, and MSI-associated immune phenotypes, and should therefore be considered when comparing results across studies.

### 3.5 Early immune engagement in premalignant SSLs

To examine how immune features vary across the serrated continuum, evidence-based immune features across HP, SSL, SSL-D, and serrated carcinoma are summarized in **Table 3**.

**Table 3:** Evidence-based immune features across the serrated neoplasia spectrum: HP, SSL, SSL-D, and serrated carcinoma.

Lesion subtype/stage	Immune-cell composition	Functional state	Spatial organization	Checkpoint/inflammatory-program evidence	Nature of evidence/key limitation
Hyperplastic polyp (HP)	Direct immune profiling remains limited; most available studies use HP mainly as a reference group rather than a deeply profiled immune subtype	No clearly established activated or exhausted immune phenotype	Spatial immune architecture is not well defined	Checkpoint-specific data are sparse at the HP level	Comparative reference category in cross-sectional pathology and single-cell studies; lesion-specific immune divergence remains under-characterized
Sessile serrated lesion (SSL)	Increased IELs and CD8^+^ cells; enrichment of CD103^+^CD8^+^ TRM, with NK and γδ T-cell signals reported in multi-omic datasets	Cytotoxic/immune-engaged features are detectable, but early regulatory programs also emerge, including Tregs and anti-inflammatory macrophage-like states	Intraepithelial/epithelial-region CD8^+^ infiltration is detectable	Early PD-1/PD-L1 upregulation can already be observed; antigen-presentation-linked inflammatory differences are reported before hypermutation	Supported by cross-sectional pathology, multi-omic, single-cell, and spatial studies; not longitudinal
SSL with dysplasia (SSL-D)	Higher IEL/T-cell infiltration than nondysplastic SSL; suppressive immune and stromal programs appear more prominent than in SSL	Coexistence of immune engagement and inhibitory regulation becomes more pronounced than in SSL	Spatial remodeling appears more evident than in SSL, but evidence remains cross-sectional	PD-1/PD-L1 expression increases further; MSI-H SSL-D tends to show stronger checkpoint-associated inflammatory features	Best-supported premalignant “transition” stage, but still inferred from different lesions/patients rather than serial evolution
Serrated carcinoma (MSI-H/dMMR-like)	Lymphocyte-rich/cytotoxic TIL-high phenotype is more often reported	Inflamed but checkpoint-rich phenotype is most consistent with available data	More infiltrated immune architecture than immune-excluded phenotypes	PD-L1 expression is associated with MSI, BRAF mutation, and cytotoxic TIL-related serrated-pathway features	Strongest support for an inflamed serrated-carcinoma endpoint, but most clinical evidence is biomarker-defined (MSI/dMMR), not origin-defined
Serrated carcinoma (MSS/pMMR, often BRAF-mutant-like)	Direct origin-annotated immune data are more limited; no equally strong evidence for a lymphocyte-rich phenotype	More compatible with mesenchymal/stromal/less immune-permissive programs than with a clearly inflamed phenotype	Immune-excluded or stromal-dominant organization is a plausible model, but direct lineage-matched proof remains limited	TGFβ-associated stromal activation provides stronger support than checkpoint-rich signaling for this state	Should be presented as a predominant trajectory/interpretive model rather than a definitively proven endpoint

This table summarizes evidence-based immune features reported across HP, SSL, SSL-D, and serrated carcinoma. Because most supporting studies are cross-sectional rather than longitudinal, the listed patterns should be interpreted as subtype- and stage-associated observations rather than direct evidence of temporal progression or causality.

Multiple studies suggest that serrated lesions already exhibit signs of immune engagement at the premalignant stage (41–43). Immunohistochemistry has shown that sessile serrated lesions (SSLs) harbor higher densities of intraepithelial lymphocytes (IELs) and CD8^+^ cytotoxic T cells than normal colonic mucosa, consistent with early immune recognition of dysplastic epithelial changes and initiation of immune surveillance (41). Single-cell transcriptomics and spatial multi-omic profiling further refine this “early surveillance” phenotype by delineating specific immune subsets and their spatial distribution (42, 43). For example, in a single-cell analysis spanning the serrated spectrum (including HP, SSL, SSL-D, and TSA), Zhou et al. reported enhanced cytotoxic gene signatures in CD8^+^ T cells within SSL samples, together with an increased proportion of CD103^+^CD8^+^ tissue-resident memory T cells (TRM), suggesting that a TRM-associated mucosal surveillance program may already be established in premalignant lesions (43).

These immune programs appear before the fully developed hypermutated MSI-H state and should therefore be distinguished from the more intense inflammatory phenotype of established MSI-H/dMMR carcinomas (41–43). Multi-omic studies similarly report increased CD8^+^ T cells, NK cells, and γδ T-cell signals in serrated polyps, with epithelial-region CD8^+^ infiltration already detectable (42, 43).

Some studies further suggest that serrated epithelium may upregulate antigen-presentation programs and chemotactic cues that facilitate local immune recruitment (42, 43). Retinoic-acid-related signaling may contribute to TRM maintenance on the basis of broader intestinal TRM biology, but this remains an indirect inference rather than lesion-specific evidence in human serrated neoplasia (46).

Premalignant SSLs do not follow a simple trajectory toward progressively stronger immune engagement. Single-cell and spatial studies indicate that regulatory T cells, suppressive macrophage-like programs, and myeloid-associated restraint may already be present and may increase with dysplastic progression, while focal reductions in CD8^+^ density have also been reported in selected spatial compartments. Overall, the available evidence indicates that premalignant SSLs already exhibit measurable immune surveillance together with emerging regulatory restraint, rather than a uniformly immune-desert phenotype (43, 63).

### 3.6 Immune checkpoint upregulation from SSL to dysplastic SSL-D

Progression from SSL to SSL-D is generally associated with increasing immune-cell infiltration and checkpoint-related regulation. In the cohort reported by Acosta-Gonzalez et al., IEL density increased from nondysplastic SSL to SSL-D and carcinoma, while MSI-H lesions showed higher IEL counts and higher lymphocytic PD-1/PD-L1 expression than MSS lesions at later stages (41). Independent cohorts have reported broadly similar trends, although effect sizes vary across studies (66).

Taken together, these findings suggest that, in stage-stratified serrated cohorts, IEL density and checkpoint expression often increase with dysplastic progression.

Checkpoint expression also tends to rise with increasing infiltration, indicating that inhibitory feedback emerges in the setting of ongoing immune engagement rather than serving as a direct marker of effective antitumor activation. In the same cohort, PD-1 and PD-L1 scores increased across progression, especially in high-grade dysplasia and carcinoma, while other studies likewise reported checkpoint upregulation during dysplastic evolution. These findings are most consistent with inflammation-associated adaptive immune regulation that may later be co-opted for immune evasion, rather than with a simple linear increase in effective antitumor immunity (67, 68).

These patterns are not necessarily contradictory. While dysplastic progression may be accompanied by increasing IEL density and checkpoint expression in some stage-stratified cohorts, the transition to invasive carcinoma may also involve spatial redistribution or exclusion of effector T cells, resulting in reduced epithelial infiltration despite ongoing immune engagement (63, 69). Taken together, this stage is particularly relevant to the proposed framework because it is where the coexistence of immune engagement and regulatory restraint becomes more clearly linked to later immune divergence.

### 3.7 Coexistence of immune activation and suppression in invasive serrated CRC

As serrated lesions progress from a premalignant state to invasive carcinoma, the tumor immune microenvironment undergoes more prominent spatial remodeling (42, 63). A quantitative study using multiplex immunofluorescence reported that, in premalignant lesions, the infiltration densities of CD3^+^CD4^+^ and CD3^+^CD8^+^ T cells were higher than those in invasive carcinoma in both the intraepithelial and lamina propria compartments (63). Upon transition to invasive cancer, T-cell infiltration showed a tendency to decrease or redistribute in overall abundance and/or spatial localization (63, 70). These observations suggest that immune exclusion may become more prominent during tumor progression, limiting the access of effector T cells to the tumor epithelium and thereby constraining cytotoxic activity (44, 63).

More granular immune phenotyping further indicates important differences in T-cell composition and functional state between premalignant lesions and invasive tumors. In one analysis, the density of CD3^+^CD8^+^FOXP3^low cells in the lamina propria was markedly higher in premalignant lesions than in the stromal compartment of invasive cancers, whereas the proportion of FOXP3^high cells (conventional regulatory T cells) did not differ substantially between the two settings (63). This pattern suggests that the major limitation of anti-tumor immunity in invasive disease may not be driven primarily by a large expansion of Tregs, but may instead reflect loss and/or spatial restriction of activated or effector CD8^+^ T cells (63, 70). Consistent with this interpretation, Ki-67–based analyses have shown a higher fraction of proliferating, activated T cells already present in serrated premalignant lesions, indicating that immune activation may be relatively robust at earlier stages (63).

With sustained exposure to immune selective pressure, immunosuppressive populations—including Tregs, M2-like macrophages, and myeloid suppressor programs—can become progressively enriched and establish physical and biochemical barriers that further limit effective CD8^+^ T-cell function (43, 54). This process can be interpreted within the framework of cancer immunoediting (elimination–equilibrium–escape) (71, 72). In serrated pathway–associated tumors, particularly within the MSI-H/dMMR trajectory, strong immune pressure may also promote the accumulation of genetic immune-evasion events, such as B2M inactivation leading to reduced MHC-I expression, or mutations affecting the IFN-γ–JAK/STAT axis that blunt tumor responsiveness to inflammatory signaling (52, 54, 73). These alterations are commonly implicated in primary or acquired resistance to immune checkpoint blockade (73–75). In this sense, invasive serrated CRC represents the stage at which the divergent immune endpoint states proposed in this review become most clinically and translationally apparent, even though substantial heterogeneity still remains.

## 4 Immunotherapy implications and translational perspectives

The translational relevance of this framework lies not in using serrated origin alone to guide treatment today, but in testing whether immune context can refine biomarker hypotheses and rationalize different therapeutic strategies for sporadic MSI-H disease and BRAF-mutant/MSS disease (45, 58, 76, 77).

### 4.1 Immune checkpoint blockade in MSI-H/dMMR serrated pathway–associated CRC

Clinical evidence for immune checkpoint blockade in this setting is established primarily at the level of MSI/dMMR rather than serrated origin. Trials such as KEYNOTE-177 and CheckMate-142 stratified patients by MSI/dMMR status, so any additional predictive role of serrated ancestry, MLH1 methylation-associated origin, or BRAF-defined lineage remains indirect and insufficiently validated (76, 77).

Within biomarker-defined MSI-H/dMMR colorectal cancer, pembrolizumab and nivolumab-based regimens have shown durable benefit in pivotal trials (76–78). However, MSI-H/dMMR CRC is itself heterogeneous: most sporadic cases arise through MLH1 methylation and often co-occur with BRAF V600E mutation, whereas Lynch-associated tumors arise through germline MMR defects (79, 80). Because most immunotherapy studies have not systematically subclassified these groups, it remains unclear whether serrated-origin sporadic MSI-H tumors differ from Lynch-related tumors in immune architecture, resistance mechanisms, or response to specific checkpoint strategies (76, 77). Emerging cohort data are beginning to address this question, but lineage-resolved evidence remains limited.

### 4.2 Therapeutic challenges and emerging strategies for BRAF-mutant/MSS serrated pathway–associated CRC

Colorectal cancer with BRAF V600E mutation and microsatellite stability (MSS) is commonly regarded as one of the most prognostically unfavorable and therapeutically challenging molecular subtypes often associated with the serrated pathway (81–83). Although these tumors can arise from serrated lesions, the lack of a high tumor mutation burden and abundant neoantigens is associated with low overall response rates to ICI monotherapy (83, 84). The phase III BEACON CRC trial established the role of combined BRAF inhibition with EGFR inhibition (with or without MEK inhibition) in previously treated BRAF V600E–mutant metastatic CRC; however, the overall survival benefit remains on the order of months, suggesting that targeting MAPK signaling alone is often insufficient for durable disease control (85–87).

Mechanistically, BRAF-mutant/MSS tumors may sustain an immune-cold or immune-excluded state through at least two complementary routes. First, a myeloid suppressive barrier can develop: persistently active BRAF V600E/MAPK signaling may upregulate chemokine networks, including CXCR2 ligands, thereby recruiting neutrophil-like polymorphonuclear myeloid-derived suppressor cells (PMN-MDSCs) and establishing an immunosuppressive front at the tumor–immune interface (55, 88, 89). Second, tumor-intrinsic immunogenicity may be reduced: MAPK pathway activation can downregulate components of antigen processing and presentation and attenuate IFN-γ–driven inflammatory feedback, limiting effective immune recognition and amplification of anti-tumor responses (54, 90, 91).

Notably, MAPK pathway blockade in this subtype may also exert an “immune-sensitizing” effect. Because feedback reactivation of EGFR signaling can attenuate the efficacy of BRAF inhibition in colorectal cancer, combined BRAF and EGFR inhibition is used to achieve more sustained suppression of MAPK pathway output (85, 86). In this setting, MAPK suppression may help counteract suppressive myeloid cells and partially alleviate immune exclusion (55, 89). It may also help restore IFN-γ-associated transcriptional programs and antigen-presentation capacity, thereby creating a more permissive microenvironment for subsequent checkpoint blockade (45, 90). Consistent with this mechanistic rationale, early exploratory studies in BRAF V600E–mutant/MSS metastatic CRC have reported signals suggesting that a triplet strategy—BRAF inhibition plus EGFR inhibition plus PD-1 blockade—may achieve higher response activity than historical targeted approaches (58). However, evidence remains largely derived from small cohorts, and the efficacy–safety boundaries require confirmation in prospective randomized trials (45, 58).

## 5 Discussion

### 5.1 Biologic and translational implications of the immune–serrated lesion linkage

Linking serrated lesions with changes in the immune microenvironment matters because it moves this review from description to interpretation: immune context becomes a candidate explanation for progression heterogeneity, a source of biomarker hypotheses, and a basis for rational translational stratification (35, 42, 43). Its significance is therefore conceptual and hypothesis-guiding rather than immediately practice-changing.

To avoid overstating translational readiness, translational implications in this setting are best stratified according to the maturity of supporting evidence, ranging from established clinical benefit to early clinical signal and preclinical or hypothesis-generating rationale.

#### 5.1.1 Practice-informing/practice-changing clinical evidence

The strongest practice-informing clinical evidence remains immune checkpoint blockade in biomarker-defined MSI-H/dMMR colorectal cancer. In this context, the main value of serrated-pathway research is interpretive rather than practice-defining: it may help refine biomarker hypotheses by integrating pathway of origin with MSI status, driver alterations, and stromal–immune features, but it does not yet define a validated therapeutic stratification framework (76–78).

#### 5.1.2 Early clinical signal

A second level of translational relevance is supported by early clinical signals, particularly in BRAF V600E-mutant/MSS CRC, a subgroup with poor conventional outcomes and frequent biological links to the serrated pathway (27, 86). In this setting, serrated pathway research provides a rationale for combination strategies designed to overcome immune resistance, including co-targeting PD-1 with pathways such as TGF-β or IL-8/CXCR2, as well as targeted therapy–immunotherapy approaches intended to convert immune-excluded tumors into more permissive states (44, 45, 58, 84). These approaches are promising, but they should still be viewed as emerging strategies supported mainly by mechanistic rationale and early clinical observations rather than practice-changing evidence (45, 58, 84).

#### 5.1.3 Preclinical/hypothesis-generating directions

Other proposed applications remain predominantly preclinical or hypothesis-generating. These include immune interception or immunoprevention in high-risk groups enriched for serrated lesions, such as serrated polyposis syndrome, preventive vaccination against antigens expressed in premalignant lesions, periodic immunomodulatory approaches aimed at reducing lesion burden or delaying progression, and metabolic targeting of vulnerabilities such as cholesterol biosynthesis (14, 92–95). Although immunoprevention has shown general feasibility in other high-risk settings, including advanced adenoma and Lynch syndrome, no dedicated immunoprevention trials have yet been reported for SPS. Likewise, vaccines, adoptive cell therapies, bispecific antibodies, and metabolism-directed strategies in serrated pathway–associated disease should currently be framed as research directions requiring further validation of biological relevance, feasibility, safety, and patient selection (14, 96–100).

### 5.2 Competing models and unresolved questions

Although the stage-linked framework proposed here is useful for integrating the available literature, it should be interpreted alongside competing explanations. One possibility is that later immune divergence is driven predominantly by epithelial evolution itself, particularly the acquisition of dMMR/MSI-H status, increased mutational burden, and neoantigen generation, with premalignant immune alterations serving mainly as accompanying rather than determining features (47, 101). A second possibility is that stromal remodeling, TGF-β-associated programs, and myeloid-barrier formation are stronger determinants of immune exclusion than serrated ancestry itself (44, 53, 88). A third interpretation is that immune evolution along the serrated pathway may be better represented as a continuum with overlapping and transitional states, rather than a discrete bifurcation (42, 43).

Key unresolved questions include whether premalignant immune changes are causal or merely correlative, whether MLH1 methylation marks or drives immune divergence, which biomarkers best capture clinically relevant immune states within BRAF-mutant/MSS disease, and whether serrated-origin biology independently modifies benefit from immune checkpoint blockade beyond established biomarkers such as MSI/dMMR (35, 42, 76, 78). Addressing these questions will require longitudinal cohorts, standardized pathology and MSI annotation, and integrative spatial and functional studies.

### 5.3 Conclusion

This review supports a cautious but important conclusion: changes in the immune microenvironment in serrated lesions may help explain why serrated-pathway cancers do not progress toward a single biologic endpoint. The current value of this framework is therefore not to change clinical management now, but to sharpen future work on progression biomarkers, lineage-resolved immune evolution, and mechanism-based therapeutic strategies.
